# Gemcabene, a first-in-class lipid-lowering agent in late-stage development, down-regulates acute-phase C-reactive protein via C/EBP-δ-mediated transcriptional mechanism

**DOI:** 10.1007/s11010-018-3353-5

**Published:** 2018-04-11

**Authors:** Rai Ajit K. Srivastava, Joseph A. Cornicelli, Bruce Markham, Charles L. Bisgaier

**Affiliations:** 1 Gemphire Therapeutics Inc, Livonia, MI USA; 2 Charles River Laboratories International, Wilmington, MA USA; 3 Diapin Therapeutics, Ann Arbor, MI USA

**Keywords:** Gemcabene, C-reactive protein, Inflammation, Atherosclerosis, C/EBP, NF-κB

## Abstract

Inflammation plays a key role in setting the stage leading to atherosclerosis progression, and high-sensitivity C-reactive protein (CRP) has been recognized as a predictor of cardiovascular risk. As a monotherapy and in combination with statins, gemcabene markedly reduced CRP in humans. Present investigation was undertaken to understand the mechanism of CRP reduction. In human hepatoma cells, gemcabene inhibited IL-6 plus IL-1β-induced CRP production in a concentration-dependent manner, reaching 70% inhibition at 2 mM. In TNF-α-stimulated primary human coronary artery endothelial cells, both CRP and IL-6 productions were reduced by 70% at 2 mM gemcabene concentration. To investigate the mechanism of gemcabene-mediated reduction of CRP, transfection studies were performed with human CRP regulatory sequences in luciferase/β-gal system that showed 25-fold increase in IL-6- and IL-6 plus IL-1β-stimulated CRP transcription. Luciferase activity was reduced by 50% by gemcabene, suggesting transcriptional down-regulation of CRP. Site-directed mutagenesis of human CRP promoter revealed that the overlapping downstream C/EBP and NF-κB binding sites are important for gemcabene-mediated CRP transcription. Gel shift assays identified the transcription factor that binds to the downstream CRP promoter as C/EBP-δ. In conclusion, gemcabene decreases CRP by C/EBP-δ and NF-κB-mediated transcriptional mechanism and suppresses IL-6 and IL-1β-induced CRP production.

## Introduction

Cardiovascular disease (CVD) is the number one cause of mortality in the Western world. Studies over the last 40 years suggest that inflammation plays an important role in setting the early stage for CVD as well as enabling the progressive development of disease allowing the further deposition of arterial lipid (reviewed in [1–4]). Interaction of immune cells with metabolic risk factors initiates and promotes lesions in the vascular smooth arterial wall [5]. These immune cells, in the presence of cytokines and inflammatory stimulants, can lead to vascular inflammation, and transform into monocyte-derived macrophages accumulating in the subendothelial space and progression of atherosclerosis [3, 6–9] through stimulation of vascular cell adhesion molecule-1 (VCAM-1), intercellular adhesion molecule-1 (ICAM-1), and endothelial cell selectin (E-selectin) [8, 9]. The chemoattractant cytokine, monocyte chemoattractant protein-1 (MCP-1), recruits monocytes to the arterial endothelium and facilitates their entry in the subendothelial space [9, 10].

Clinical outcome studies over the last 15 years strongly support the inflammatory nature of atherosclerosis [11–13]. Several other studies have linked inflammatory biomarker, high-sensitivity C-reactive protein (CRP), with CVD risk [14–17]. CRP is an acute-phase reactant released during the inflammatory processes [18, 19], and is recognized as a powerful predictor of cardiovascular risk [20–22]. Statin therapy not only lowers low-density lipoprotein (LDL) cholesterol levels, but also reduces CRP [23, 24]. Thus, the magnitude of risk reduction associated with statin therapy may exceed that which is expected just on the basis of the LDL-C lowering. Prospective evidence provided by the JUPITER trial (justification for the use of statins in primary prevention: an intervention trial evaluating rosuvastatin) demonstrated that rosuvastatin-treated patients with normal LDL-C levels, but elevated baseline CRP levels, showed highly significant (− 44%) reduction in adverse cardiovascular events [14, 19], suggesting additional benefits of CRP reduction, and further demonstrating an inflammatory component in CVD risk [16]. Notably, either previous or early initiation of CRP lowering by statin therapy, after an ischemic stroke, improves the survival and readmission rates [25]. Thus, CRP is a marker of inflammation and atherosclerosis that may play an active role in the atherogenic process [26]. In patients with acute coronary syndrome, CRP released in the coronary circulation has been found to cause endothelial dysfunction [27], possibly through dissociation of complex between CRP and lysophosphatidylcholine [28].

Gemcabene calcium (also known as gemcabene, CI-1027 and PD 72953-0038) is a small molecule and is the monocalcium salt of a dialkyl ether dicarboxylic acid with the chemical name 6,6′-oxybis (2,2-dimethylhexanoic acid) monocalcium salt, and is currently in late-stage clinical development. Gemcabene has shown hypolipidemic properties in preclinical [29] and clinical [30, 31] studies. In addition to cholesterol lowering across all studies, gemcabene lowers plasma CRP levels in patients by as much as 53.5% alone, and by as much as 71% with statins indicating that this compound has anti-inflammatory properties. This was further corroborated in a recent clinical study [31]. Since CRP is produced mainly in the liver in response to interleukin-6 (IL-6) [32], we evaluated the effect of gemcabene on CRP production by cytokine-stimulated human hepatoma PLC/PRF/5 (Alexander) cells. We studied IL-6 and IL-1β-induced inhibition of CRP by gemcabene since IL-6 and IL-1β are responsible for acute-phase induction of CRP [33]. The IL-6 response is mediated through the CCAAT-enhancer-binding protein (C/EBP) in the proximal promoter, while the IL-1β response appears to be regulated through an NF-κB response element [33]. The latter site has been shown to mediate CRP down-regulation in response to fibrates in HuH7 hepatoma cells [34]. This effect of fibrate was also observed in dyslipidemic and diabetic patients [35–37].

In the current study, we show that the CRP promoter is up-regulated by IL-6/ IL-1β or with IL-6 alone in a human hepatoma cell line. This induced activity is inhibited by gemcabene and this inhibition is dependent on the presence of a functional downstream C/EBP response element. Gemcabene had no effect on basal expression of CRP. Our findings also suggest that the NF-κB response element may influence gemcabene-mediated inhibition of transcription; however, these results were not conclusive. Finally, the STAT sites in the proximal human CRP promoter do not appear to be required for the gemcabene-mediated inhibition of CRP promoter activity.

## Materials and methods

### Materials

The human hepatoma cell line PLC/PRF/5 (Alexander) (ATCC CRL-8024, American Type Culture Collection, Manassas, Virginia) was maintained in Minimum Essential Medium Eagle (MEM) (Cat. No. 30-2003 ATCC, Manassas, Virginia) supplemented with 10% fetal bovine serum (Cat. No.16000-044 Gibco, Grand Island, New York). Dexamethasone was purchased from Sigma, (Cat. No. D-8893, St. Louis, Missouri). IL-6 and interleukin-1 beta (IL-1β) were purchased from R&D System (Cat. No.206-IL-010, 201-LB005, Minneapolis, Minnesota) and CRP Elisa Kits were purchased from Alpha Diagnostic International Inc. (Cat. No.1000, San Antonio, Texas). DC Protein Assay Kit was purchased from Bio-Rad Lab (Cat. No 500-0116, Hercules, California).

### Cell culturing and drug treatments

PLC/PRF/5 (Alexander) human hepatoma cell monolayers were grown to confluence (6 days following splitting) into 6-well tissue culture plates. Cells were washed 3 times with pre-warmed medium and then treated with 1 mL of medium with or without varying concentrations of gemcabene. After 1 h, the medium was replaced with fresh medium containing cytokines (10 ng/mL IL-6 and 1 ng/mL IL-1β), 1 µM dexamethasone, and gemcabene at the concentrations indicated. After 24 h of incubation, the medium was collected and centrifuged for 5 min at 1000 rpm at room temperature. Supernatants were collected and frozen for analysis of CRP. Cells were also used for total cell protein measurements.

### Cell culture conditions

The protocols for culturing Alexander cells and for inducing CRP production with IL-6 have been reported previously [33]. Briefly, Alexander cells were cultured in MEM, supplemented with 10% FBS and 100 µg/mL streptomycin sulfate-100 U penicillin G. For CRP assays, cells were trypsinized and seeded in 96-well cell culture plates at 12,000 cells per well in 200 µL growth medium and allowed to grow for 5 days. The evening prior to the experiment, the cells were treated with dexamethasone (10 nM) in order to induce a differentiated state. Subsequently, the medium was changed to MEM supplemented with 0.2% BSA for the assay.

Human coronary aortic endothelial cells (HCAEC) (Cambrex CC-2585) were grown according to the protocol supplied with Clonetics Coronary Artery Endothelial Cell System kit, which included an optimized growth medium, EGM-2-MV BulletKit (Cambrex CC-3202). The kit included a basal medium-2 and several growth supplements as well as a trypsin, trypsin-neutralizing solution, and HEPES-buffered saline solution. HCAEC cells can be grown for about 7 passages. Deterioration in growth rate and biological responsiveness were noted with additional passages. The growth medium was changed every other day and cells were passaged when they reached 60–80% confluence. For the assay, the cells were seeded at 3200 cells per well in 96-well plates and allowed to grow until confluent.

### CRP assay

The Alexander cells were treated with gemcabene for 24 h at concentrations indicated in respective figure legends. The final concentration of DMSO in the assay medium was 1%. Following the pre-incubation, 200 µL of fresh media (R&D Systems 201-LB) with compound and IL-6 and IL-1β were added to the wells for a final concentration of 30 and 1 ng/mL, respectively. Cells were incubated at 37 °C for an additional 24 h. Medium was removed from each well and transferred to new 96-well plates. Media samples (10 µL) and antibody conjugate (100 µL) were added to CRP ELISA plates (Alpha Diagnostic International 1000) and the plates were sealed in plastic wrap and incubated overnight at 4 °C. The wells were washed with 200 µL dH_2_O, and substrate mix (200 µL kit component) was added to each well, mixed gently, and incubated at room temperature for 10 min. The reaction was stopped with 50 µL of Stop Solution (kit component). The absorbance was measured at 450 nm using a multiplate spectrophotometer (Molecular Devices, Spectra MAX Plus with SoftMax Pro software). The amount of CRP is calculated against a CRP standard curve.

### Human IL-6 assay

HCAECs were pre-incubated with gemcabene for 24 h at concentrations indicated in the figure legends, with final concentration of DMSO in the assay medium of 0.2%. Following the pre-incubation, 200 µL medium with gemcabene and rhTNF-α, 10 ng/mL final concentration, (R&D Systems, Minneapolis, MN, Cat # 210TA010), were added and cells were incubated at 37 °C for an additional 24 h. Subsequently, the medium was removed from each well and transferred to new 96-well plates. Media samples (100 µL) were added to assay diluent (100 µL) in human IL-6 ELISA plates (R&D Systems D6050) and incubated at RT for 2 h. The plates were then washed four times with 200 µL of wash buffer (kit component), 200 µL of antihuman IL-6 conjugate was added, and the plates were incubated for 2 h at room temperature. The plates were then washed four times with 200 µL of wash buffer and 200 µL of substrate was added. After 20 min, the reaction was stopped with 50 µL of 2 N sulfuric acid, mixed, and read in a multiplate reader at 450 and 570 nm, and the absorbance at 570 nm were subtracted from that at 450 nm. This number was compared to the standard IL-6 curve to give pg/mL.

### Cell viability assays

For lactate dehydrogenase (LDH) levels (Promega Cytotoxicity Assay Kit G1780, Madison, WI), 50 µL of medium from each well was assayed with 50 µL of substrate in covered plates for 30 min at room temperature. The reaction was stopped with 50 µL of stopping solution, mixed, and read in a multiplate reader at 490 nM. In some experiments, to measure total cell protein, the cells were washed with 200 µL PBS and lysed with 100 µL Promega reporter lysis buffer, and 25 µL of the lysate was assayed for protein using the Pierce BCA protein assay reagent kit (Waltham, MA) utilizing BSA as the standard. The plates were read in a multiplate spectrophotometer (Molecular Devices, Spectra MAX Plus with SoftMax Pro software, Sunnyvale, CA) and the absorbance was compared to the BSA standard curve to derive protein concentration.

### Total cell protein measurements

Another method was used to measure cell protein in some experiments. After removal of the media, the cells were used for total cell protein measurements as follows. One mL of 0.1 N NaOH was added to the cells in each well and the mixture was frozen at − 20 °C overnight. The following day, the cell lysate was harvested, and protein concentrations were determined using a DC Protein assay kit (Bio-Rad, Hercules, CA). Bovine Serum Albumin reference standard (10 µL) or cell lysate (10 µL) was pipetted into a cell of a Microplate. Reagents A (25 µL) and Reagent B (200 µL) were then added to each well (Reagent A and B are as described by the manufacturer of the DC Protein assay kit) and incubated at room temperature for 15 min. At the end of this incubation, absorbance was measured at 690 nm in a Spectra Max Plus Molecular Devices spectrophotometer.

### Preparation of nuclear extracts

PLC/PRF/5 cells were treated with dexamethasone (10 nM) for 16 h, followed by a 2-h pretreatment with or without gemcabene (2 mM). Cells were then incubated with IL-6 (30 ng/mL) and harvested for nuclear extract preparation at different time points up to 24 h. Nuclear extracts were prepared using a Nuclear Extract Kit (Cat. # 40010, Active Motif, Carlsbad, California) according to the manufacturer’s instructions. Nuclear protein concentration was determined using Bio-Rad reagent with BSA as a protein concentration standard. Nuclear extracts were stored at − 80 °C in 10 µL aliquots until use.

### Annealing and labeling of oligonucleotides

All the gel shift oligonucleotides used in the current study are shown in Table 1. Particularly, the two known C/EBP binding sites on the CRP promoter (C/EBPup and C/EBPdown binding sites) were used as DNA probes for binding assay. A 22 bp double-stranded oligonucleotide containing the C/EBPup binding site, from − 245 to − 223 in the CRP promoter, as well as a 19 bp double-stranded oligonucleotide containing the C/EBPdown binding site, from − 77 to − 59 in the CRP promoter were used as probes. Mutant competitive oligonucleotides were synthesized containing the mutations described in Table 1. The C/EBP consensus and mutant oligonucleotides were purchased from Santa Cruz Biotechnology (Santa Cruz, CA 95060). Two complementary oligonucleotides were annealed in an annealing buffer (67 mM Tris–HCL pH 7.6, 13 mM MgCl_2_, 6.7 mM DTT, 1.3 mM spermidine, 1.3 mM EDTA) at 85–90 °C for 5 min in a heat-block and then the heat-block was removed from the heating unit and allowed to cool to 35 °C. The annealed double-stranded oligonucleotides were then used for end-labeling or as cold competitors. To prepare radiolabeled probe, double-stranded oligonucleotides (10 pmol) were labeled by end-labeling method, using 50 µCi of ^32^P-ATP (Amersham Biosciences, Piscataway, NJ 08855-1327, USA) Cat No. PB10218, 6000 Ci/mmol) and 2 units of T4 Polynucleotide Kinase (Roche Diagnostics, Indianapolis, IN 46250-0457) at 37 °C for 30–60 min. A G-25 Sephadex spin column was used to purify this labeled ds-oligonucleotide.

**Table 1:** Oligonucleotides used for CRP promoter analysis

Upstream C/EBP			
Forward	5′	CAA AGT GGA GCC CTG AGA TAT TTA TTC ATT TTT CCT GTC	3′
Compliment	5′	GAC AGG AAA AAT GAA TAA ATA TCT CAG GGC TCC ACT TTG	3′
Downstream C/EBP			
Forward	5′	GAA AAT TAT TTA CAT AGT GGC TCC AAC TCC CTT ACT GC	3′
Compliment	5′	GCA GTA AGG GAG TTG GAG CCA CTA TGT AAA TAA TTT TC	3′
NFKB			
Forward	5′	CAT AGT GGC GCA AAC GAT ATT ACT GCT TTG GAT ATA AAT CC	3′
Compliment	5′	GGA TTT ATA TCC AAA GCA GTA ATA TCG TTT GCG CCA CTA TG	3′
NFKB with downstream C/EBP			
Forward	5′	CAT AGT GGC TCC AAC GAT ATT ACT GCT TTG GAT ATA AAT CC	3′
Compliment	5′	GGA TTT ATA TC AAA GCA GTA ATA TCG TTG GAG CCA CTA TG	3′
STAT site 1^a^			
Forward	5′	CAG TAG TCA TAG GAG TCC GTA ATA AAT AAC TCA C	3′
Compliment	5′	GTG AGT TAT TTA TTA CGG ACT CCT ATG ACT ACT G	3′
STAT site 2^a^			
Forward	5′	CAT TGA TTT CTC TGC CCT GAA ATA ATT TTG	3′
Compliment	5′	CAA AAT TAT TTC AGG GCA GAG AA TCA ATG	3′
STAT site 3^a^			
Forward	5′	GCT TCC CCT CCC CCC GAA GCT CTG ACA CAC C	3′
Compliment	5′	GGT GTG TCS GAG CTT CGG GGG GAG GGG AAG C	3′
STAT site 4^a^			
Forward	5′	CTG CCC CAA CAA GCA ATG CCG GAA AAT TAT TTA C	3′
Compliment	5′	GTA AAT AAT TTT CCG GCA TTG CTT GTT GGG GCA G	3′

For the NF-κB mutations, the primers were based on sequences described previously. PCR reactions were set up using reagents from the mutagenesis kit, the oligos (20 pM), and mini-prep DNA (1:20 dilute) of the clone to be mutated. Oligos were purified by polyacrylamide gel electrophoresis (PAGE). Efforts were made to have > 40% GC content, a Tm around 78 °C, ending the oligo in G’s and C’s where possible

^a^STAT site number refers to their sequential order in the gene

### Gel shift assay

Gel shift assays were carried out in a reaction mixture containing 10 µg of nuclear extract protein, 2 µL of 10× binding buffer (200 mM Tris–HCL pH 7.6, 500 mM KCL, 10 mM MgCl_2_, 10 mM DTT, 2 mM EDTA, 0.1% (v/v), Triton X-100, 50% (v/v) Glycerol, and 5 mM spermidine), 2.5 µL poly dI·dC (1µg/L), and nuclease-free water in a total volume of 19 µL. After 10-min incubation at room temperature, 1 µL of ^32^P-labeled oligonucleotides (40 fmols, approximately 50,000 cpm) was then added to the reaction and incubated at room temperature for 45 min before loading to the gel. The resulting DNA–protein complex was separated from free probe by electrophoresis on a 6% polyacrylamide gel with 0.5× TBE buffer. For binding competition analysis, non-radioactive oligonucleotides (50×–100× molar excess) were added as competitors to the binding reactions as indicated in the figure legends (Table 1). For antibody interaction studies, antibodies against C/EBP-α, C/EBP-β, C/EBP-γ, C/EBP-δ, and PPAR-α (Santa Cruz Biotechnology, Santa Cruz, CA 95060) were added into the reaction mixture during the pre-incubation time. The electrophoresis was performed at 4 °C, 240 V for 2.5 h. The gel was then dried and visualized following autoradiography for 16 h at − 80 °C without an intensify screen. The densitometry quantification was performed using a Bio-Rad Image Densitometer (Model GS-700).

### Cloning and mutation of the CRP promoter

The human CRP promoter clone was isolated from human genomic DNA (Clontech, Mountain View, CA) by PCR amplification using oligonucleotides (Table 1) containing the restriction sites Nhe I and Xho I. The sequence was derived from the reported sequence [33, 38]. The amplified product was cloned into the TA cloning vector pCRR2.1 (Invitrogen, Waltham, MA). The TA cloning vector was subsequently digested with NheI and XhoI and the CRP promoter fragment was sub-cloned into pGL3 basic (Promega) at the Nhe I and Xho I sites. The resulting clone, designated pCRP900, contains CRP sequences from position 765 to 1750 in the GenBank accession number AF449713 corresponding to − 976 to + 9 with respect to the transcription start site (+ 1). The clone was sequenced to confirm its identity. Site-directed mutations were introduced into the previously described C/EBP binding sites, the NF-κB site, and potential STAT sites located in the proximal promoter. Mutations were introduced using Stratagene’s (Kirkland, WA) QuikChange Site-Directed Mutagenesis Kit according to the basic protocol, with a few modifications.

Oligonucleotides were designed using the guidelines set forth in the Stratagene protocol; 15–18 nt on each side of the mutations, *a* > 40% GC content, a *T*_m_ around 78 °C, ending the oligo in G’s and C’s where possible, and having the oligo PAGE purified. For the NF-κB mutations, the primers were based on sequences described previously. PCR reactions were set up using reagents from the mutagenesis kit, the oligos (2 CM), and mini-prep DNA (1:20 dilute) of the clone to be mutated.

For a single reaction, the following components were added in a total volume of 100 µL:10 µL Reaction Buffer Mini-prep DNA (1:20); Forward Oligo (20 mM); Compliment Oligo (20 mM) dNTP; Taq DNA polymerase. The total volume of 100 µL was made with deionized sterile water. After the reagents were added, they were mixed gently and centrifuged to bring down the mixture. Approximately, 5 µL of mineral oil was placed on top of each reaction tube. The tube was placed into the thermal cycler which was heated to 95 °C for 30 s and then run for 18 cycles at 95 °C for 30 s (strand separation), 55–58 °C for 30 s (annealing), and 68 °C for 2 min/kb (extension). Finally, the reactions were held at 68 °C for 10 min to allow for completion of extensions. When the reaction was complete, it was cooled down to 4 °C, then 1 µL of Dpn I was added to the PCR products below the mineral oil. This was mixed and spun down. The digestion mixture was incubated at 37 °C for 70–90 min. After digestion, the products were placed into a new tube and 10 µL was transformed into DH5 cells (100 µL) and transformants were selected on LB-Ampicillin (LB-AMP) plates containing 100 µg/mL ampicillin. The resulting clones were then grown, purified, and sequenced to confirm that the desired mutations were present. The C/EBP and the NF-κB mutations were each made into individual clones and in combinations, combining the C/EBP mutations with or without the NF-κB mutations in clones. The CRP promoter and mutations introduced into the promoter are shown schematically in Fig. 4A.

### Cell culture and transfection

Alexander cells were seeded at 1 × 10^5^ cells/well in a 24-well plate and grown for 48 h in MEM supplemented with 10% FCS and antibiotics. The cells were then fed with antibody-free medium. After a 24 h incubation, the medium was changed to serum-free Opti-MEM (0.5 mL) (Gibco, Big Cabin, OK 74332) and cells were transfected with 0.5 µg of wild-type or mutant CRP promoter constructs and 0.0125 g of pCMV β-gal using lipofectamine 2000 (Invitrogen) according to the manufacturer’s instructions. After 4 h, 0.5 mL of complete medium was added to the transfection mixture and the cells were incubated overnight. The following day the medium was changed, and cells were grown in 1.0 mL of complete medium overnight. In the afternoon of the next day, the medium was changed and supplemented with 10 nM dexamethasone and the cells were incubated overnight.

### CRP induction assay

On the day of CRP induction, cells were incubated 0.5 mL of Opti-MEM supplemented with 0.2% BSA, 1% DMSO. Cytokines, IL-6 (30 ng/mL) and IL-1β (1 ng/mL) or IL-6 (30 ng/mL) alone was added and the cells were incubated at 37 °C for 24 h. The cells were then harvested and assayed for luciferase and β-gal activity as described below.

### Luciferase and β-gal activity assays

Luciferase and β-galactosidase were assayed using the Dual-Light® System (Applied Biosystems, Carlsbad, California 92008) according to the manufacturer’s instructions and measured on a MicroLumatPlus LB 96 V (EG&G Berthold, Oak Ridge, TN 37830). The fold change represents the ratio of relative light units/β-gal activity of the treated groups versus the untreated control, which was set to 1.

### Data analysis

Data were analyzed by GraphPad Prism (La Jolla, CA) statistical program and significant differences between treatments were determined by either the *Z*-factor for large data sets or ANOVA as described in the literature.

## Results

### Gemcabene inhibits cytokine-induced CRP production in Alexander cells

**Fig. 1:**
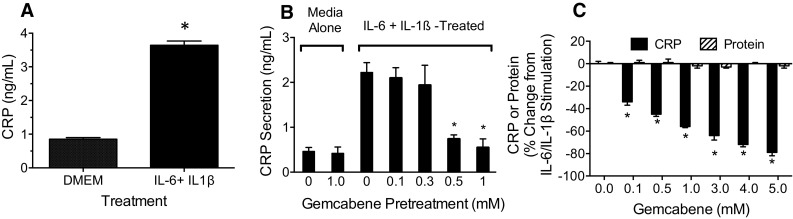
Cytokine-Induced CRP Production in PLC/PRF/5 Alexander Human Hepatoma Cells. **A** PLC/PF5/Alexander cells were grown in culture medium and assay was performed as described in “Materials and methods” section. The graph bar showing DMEM contained 1% DMSO and served as control. C-reactive protein (CRP) Production in PLC/PRF/5 Alexander cells treated with or without IL-6 + IL-1β. Bars represent the mean ± SEM of each treatment group. *N* = 4 in duplicate; *p < 0.0001. **B** CRP production in IL6 + IL1-β induced PLC/PRF/5 Alexander cells treated with or without gemcabene (0.1–1.0 mM). Bars represent the mean ± SEM of each treatment group (*n* = 4). Graph bar showing no gemcabene contained 1% DMSO and served as control. *p < 0.001. **C** Concentration response of inhibition of CRP secretion by gemcabene (0.1–5 mM) from Alexander human hepatoma cells induced with IL6 + IL1-β. In addition to CRP production, cellular protein and LDH were also measured. Figure shows only CRP (solid bars) and protein (hatched bars). Graph bar showing no gemcabene contained 1% DMSO and served as control. *Significantly different compared to untreated control (*p* < 0.001)

Confluent Alexander cells treated with IL-6 plus IL-1β result in a fourfold increase in CRP secretion (Fig. 1A). This is representative of a typical experiment where these conditions often induce a 4−5-fold increase in CRP. Gemcabene alone had no effect on CRP secretion (Fig. 1B). However, gemcabene pretreatment of the Alexander cells blocked the cytokine-induced CRP production in a concentration-dependent manner (Fig. 1C). We observed similar results in another hepatoma cell line, HepG2 (data not shown). These results suggest an IC_50_ between 300 and 500 µM for gemcabene- induced inhibition of CRP production. The concentrations chosen in the present cell-based study were based on the human multiple dose pharmacokinetic data showing a *C*_max_ of 0.893 M. This is consistent with cell-based studies of similar hypolipidemic small molecules like fenofibrate which had *C*_max_ of 0.031 mM, and for cell-based studies 0.1–0.5 mM drug concentrations were used [39, 40]. Similarly, 0.5–1 mM concentration of metformin was used in cell-based assay [41].

To determine if gemcabene reduction in CRP secretion was specific and unrelated to potential non-specific decrease in cell viability, Alexander cells were pre-incubated for 24 h with varying amounts of gemcabene followed by determination of CRP and LDH in media and cell protein content. Figure 1C shows that gemcabene decreased IL-6/IL-1β-induced CRP production in a dose-dependent manner, with > 60% inhibition at 2 mM gemcabene. Significant inhibition of CRP secretion was observed at all gemcabene concentrations tested, however at 1.5 and 2 mM gemcabene, LDH levels (not shown) in the media increased despite no change in total cell protein. These data suggest an increase in cell membrane permeability without a decrease in cell viability. In other experiments, the LDH levels in the medium remained unchanged; however, gemcabene pretreatment times were limited to 2 h (Fig. 1C). These results indicate that gemcabene inhibits CRP production at concentrations where the cells remain viable.

### Gemcabene inhibits TNF-α-induced IL-6 production in HCAEC

**Fig. 2:**
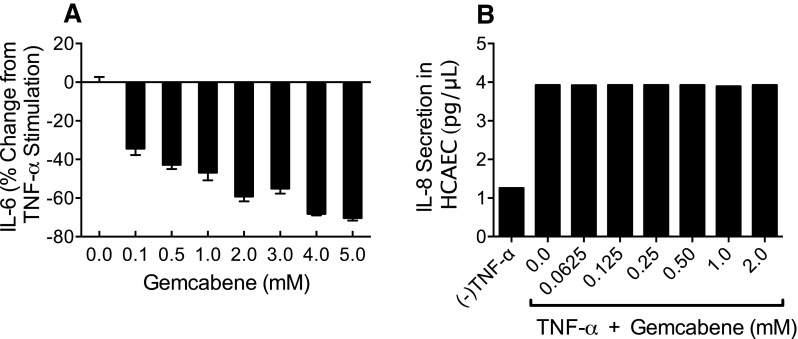
Concentration response of Gemcabene Inhibition of IL-6 Production from TNF-α Stimulated HCAE Cells. **a** HCAE cells were treated with TNF-α as described in “Materials and methods”. Secretion of IL-6 was measured following treatment with gemcabene (0.1–5 mM) *p* < 0.001. **b** shows IL-8 secretion by gemcabene (0.0625–2 mM) in TNF-α stimulated HCAE cells. All cells showed same level of secretion as seen with TNF-α alone. Results are average of measurements of duplicate samples. While there were differences in the levels of IL-8 in the media, they are not visible in the graph because of very small differences. The control assay with no gemcabene contained 1% DMSO

Gemcabene’s anti-inflammatory effects in HCAEC were assessed in a cell type involved in atherosclerotic plaque formation. As HCAEC do not produce CRP (data not shown), we instead evaluated the effect of gemcabene on TNF-α stimulated cytokine, IL-6, secretion. It is known that TNF-α induces inflammatory cytokines and adhesion molecules from endothelial cells [42]. It is also well known that the HCAEC-secreted cytokine, IL-6, stimulates CRP [38, 43], which in turn exerts proinflammatory effect on HCAEC [44]. The assay conditions were similar to those used for the Alexander cells. HCAEC were pre-incubated for 18 h with varying gemcabene concentrations (0.1–5.0 mM) followed by stimulation with TNF-α for an additional 18 h. Gemcabene pretreatment resulted in a concentration-dependent decrease in IL-6 secretion with a 70% decrease at the maximum concentration tested (5.0 mM) (Fig. 2A). In HCAEC, the media LDH levels and total cell protein were unchanged (not shown), suggesting that cell viability was not compromised. Gemcabene appears selective since it did not inhibit TNF-α induced IL-8 secretion in these cells (Fig. 2B).

### Gemcabene inhibits CRP promoter activity in the PLC/PRF/5 human hepatoma cells

**Fig. 3:**
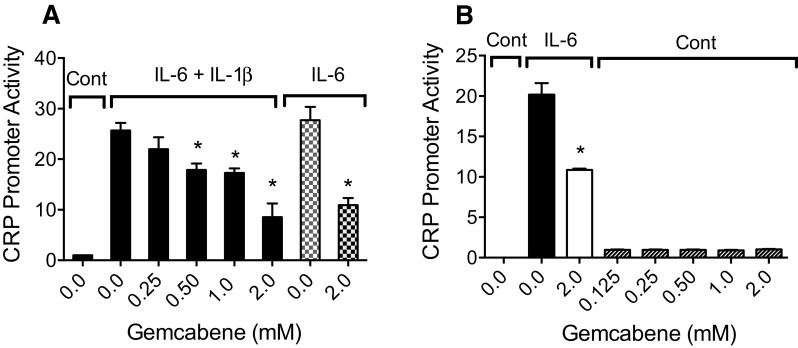
IL-6 and IL6 + IL1-β stimulated transcription from the human CRP promoter is inhibited by gemcabene. pCRP 900 was transfected into PLC/PRF/5 Alexander cells. Cells were treated as indicated according to the protocol as described in “Materials and methods”. The control assay with no gemcabene contained 1% DMSO. **A** Concentration response of gemcabene inhibition of IL-6 and IL6 + IL1-β stimulated CRP promoter activity. *Cont* control without cytokine (IL6 + IL1-β) induction. *Indicates significant difference (*p* < 0.01). **B** Lack of inhibition of basal expression from the human CRP promoter by gemcabene. Alexander human hepatoma cells transfected with pCRP900 were treated as indicated according to the protocol described in “Materials and methods”. Cells were treated with media with or without IL-6 after 2 h pre-incubation with gemcabene. *Cont* control without cytokine (IL6 + IL1-β) induction. *Indicates significant difference (*p* < 0.01)

A pCRP900 clone was constructed which contained the human CRP regulatory sequences from − 976 to + 9 relative to the transcription start site (+ 1) and was fused upstream to control the expression of a luciferase reporter gene. This pCRP900-reporter construct was transfected into Alexander cells untreated or treated with varying concentrations of gemcabene. In the absence of gemcabene or cytokine-stimulation, CRP promoter activity was minimal. A mixture of IL-6 plus IL-1β stimulated CRP expression about 25-fold over the untreated control. Gemcabene (0.25–2.0 mM) pretreatment (2 h) resulted in a concentration-dependent decrease in the cytokine-induced CRP expression (Fig. 3A). Similarly, IL-6 alone increased CRP promoter activity 25-fold. Gemcabene (2 mM) treatment inhibited IL-6-induced CRP promoter expression by greater than 50% (Fig. 3A). In a separate experiment, gemcabene (0.125–2.0 mM) alone had no effect on the basal human CRP promoter expression, although these cells were capable of responding to IL-6 and gemcabene (2 mM) as before (Fig. 3B). In both experiments (Fig. 3A, B), these cells were responsive to IL-6 and were inhibited by 2 mM gemcabene by 50%, suggesting gemcabene inhibits IL-6 induced inflammation.

### Gemcabene inhibition of IL-6 induced gene expression is mediated through C/EBP binding sites

To explore the mechanism by which gemcabene reduces IL-6-induced CRP expression, we investigated effects on CRP promoter constructs containing mutated C/EBP, NF-κB, and STAT regulatory elements [33, 45, 46]. Previous work has shown that IL-6 mediated induction of the human CRP promoter is regulated by two C/EBP response elements in the CRP proximal promoter region [45]. We carried out two types of experiments to explore the regions of the CRP promoter potentially responsible for gemcabene-mediated inhibition of CRP promoter activity. One set of experiments dealt with mutations created in the CRP promoter and measurement of promoter activity, and the other set of experiments explored the binding of the transcription factors to the promoter region.

**Fig. 4:**
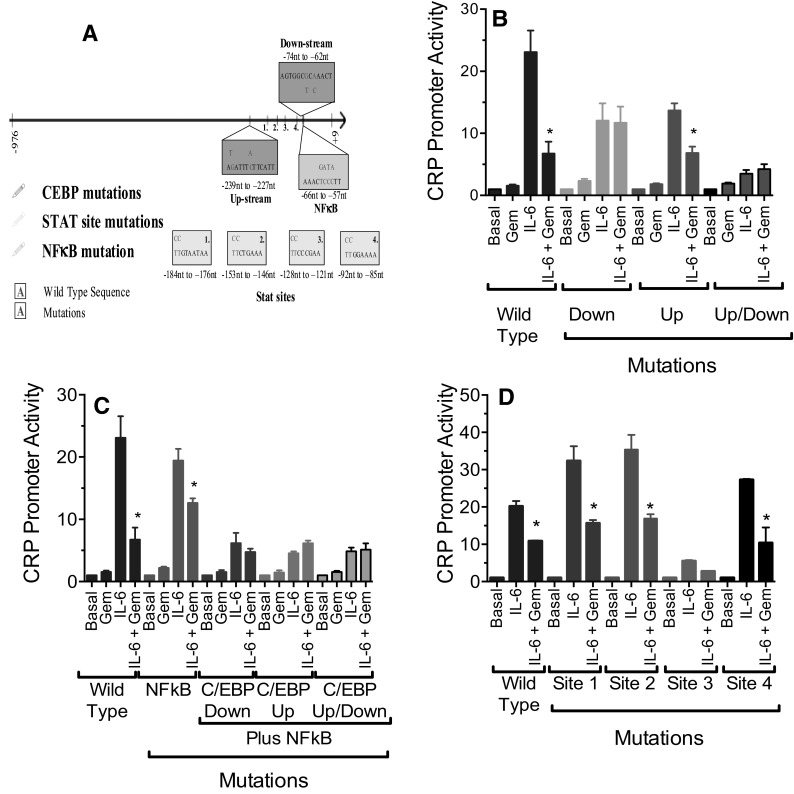
Effect of C/EBP response element mutations on IL-6 induction and gemcabene inhibition of transcriptional activity from the human CRP promoter. **A** Wild-type and mutant sequences were used in the studies to investigate CRP proximal promoter involvement in gemcabene-mediated down-regulation of CRP promoter activity. **B** Alexander human hepatoma cells transfected with pCRP900 wild-type or mutant promoter constructs and treated as indicated according to the protocol described in “Materials and methods”. Down is the downstream C/EBP mutation in pCRP900, up is the upstream C/EBP mutation in pCRP900, and up–down is the double mutant. Gemcabene concentration used was 2 mM. *Indicates significant difference (*p* < 0.01). **C** Effect of C/EBP response element mutations and NF-κB mutation on IL-6 induction and gemcabene inhibition of the human CRP promoter. *Indicates significant difference (*p* < 0.01). **D** Effect of STAT site mutations on transcription from the human CRP promoter. Alexander human hepatoma cells transfected with pCRP900 wild-type or STAT site mutant promoter constructs were treated as indicated according to the protocol described above. STAT site mutations are described in the CRP mutant diagram in (**A**). The control assay with no gemcabene contained 1% DMSO. *Indicates significant difference (*p* < 0.01)

Since gemcabene modulates IL-6-induced but not basal CRP expression, we hypothesized that inhibition required intact C/EBP response elements. Consequently, we introduced mutations into the upstream and downstream C/EBP sites either alone or in combination with each other in pCRP900 (Fig. 4A; Table 1). To study the effect of gemcabene on CRP promoter activity, wild-type and mutated promoter constructs were transfected into the Alexander human hepatoma cells followed by treatments as shown in Fig. 4B–D.

First, we evaluated the C/EBP response element. We found in the wild type, as expected, IL-6 markedly induced promoter activity which was inhibited by gemcabene (Fig. 4B). Mutation of C/EBP binding site reduced IL-6 induced CRP promoter activity by at least 50% (Fig. 4B). Further, induced activity from the upstream mutant was inhibited nearly 50% with gemcabene treatment. In contrast, gemcabene had no effect on the IL-6 induced activity of the downstream mutant. It should be noted that the upstream and downstream mutants, individually, were each induced by IL-6 albeit to a much lower level compared to the wild type. This was expected since each mutant still has 1 functional C/EBP response element. When the promoter contained mutations in both C/EBP sites (up and down), IL-6 treatment resulted in only a marginal induction of promoter activity that was not inhibited by gemcabene (Fig. 4B). These results demonstrate that IL-6-induced CRP promoter activity is regulated through the C/EBP response elements and that a functional downstream element is required for gemcabene-mediated inhibition. These results also suggest that the downstream element has distinct functional properties from the upstream element. Thus, while both C/EBP sites contribute to IL-6 induction of CRP, only the downstream site is important for inhibition by gemcabene.

### The NF-κB site that overlaps the downstream C/EBP response element may influence gemcabene inhibition of CRP

NF-κB has been shown to play a role in fibrate-mediated inhibition of IL-1β induced CRP expression [34]. The NF-κB site overlaps the downstream C/EBP binding site [34, 45]. To determine the role of the NF-κB site in gemcabene-mediated inhibition of IL-6 induced CRP activity, we introduced the same mutation in the NF-κB site as that previously reported [34] (Fig. 4A; Table 1). This mutation overlaps the downstream C/EBP site by 1 bp at − 66 nt. The mutation was also introduced into the CRP promoter in combination with the upstream and downstream C/EBP binding site mutations. These promoter constructs were transfected into the Alexander human hepatoma cells which were then treated as shown in Fig. 4C. The wild-type promoter behaved as expected based on results shown in Fig. 3, that is, gemcabene alone had no effect on CRP expression, induction occurred following IL-6 treatment, which was inhibited by gemcabene pretreatment. The NF-κB mutant construct harboring cells showed induction of promoter activity by IL-6 similar to the wild-type promoter but the percent inhibition of this activity by gemcabene was suppressed compared to that observed with the wild-type promoter (Fig. 4C), suggesting a role of NF-κB in gemcabene-mediated down-regulation of CRP transcription. Further, the NF-κB mutation in combination with the upstream mutation completely prevented the gemcabene response (Fig. 4C) seen with the upstream mutation alone (Fig. 4B). Gemcabene had no effect on activity from promoter constructs containing the downstream or upstream plus downstream C/EBP mutations in combination with the NF-κB mutation. Similar results were obtained when stimulation was induced by IL-6 and IL-1β combined (data not shown). These results are suggestive that the NF-κB site influences the effect of gemcabene on this promoter. However, because the NF-κB mutation introduced overlapped the downstream C/EBP site by a single bp, it is possible that the effects observed may have been affected by the overlapping sequence.

### STAT sites in the proximal CRP promoter do not mediate the effects of gemcabene on IL-6-induced CRP promoter expression

Between the two C/EBP response elements in the human CRP promoter, we identified four potential signal transducers and activators of transcription (STAT) response elements including one that fits a consensus for binding STAT3 [47] (Fig. 4A). Since STAT3 has been implicated as part of the IL-6 signaling pathway [43], we introduced mutations into each of the potential STAT sites to determine if they affected IL-6 induction and resulted in gemcabene-mediated inhibition. These promoter constructs were transfected into the Alexander human hepatoma cells which were then treated as shown in Fig. 4D. In terms of the IL-6 response of the CRP promoter, STAT site mutation 3 reduced the promoter activity while mutations in sites 1, 2, and 4 increased activity compared to the wild-type promoter; induction in the promoter activity observed was more in site 1 and 2 mutant constructs as compared to site 4 mutation (Fig. 4D). These results suggest that site 3 is a functional STAT site. However, the promoter containing this individual STAT site mutation could be inhibited by gemcabene by approximately 50%. This result indicates that the gemcabene effect is not mediated through these potential STAT sites.

### Identification of proteins from nuclear extracts prepared from Alexander human hepatoma cells that interact with C/EBPup

**Fig. 5:**
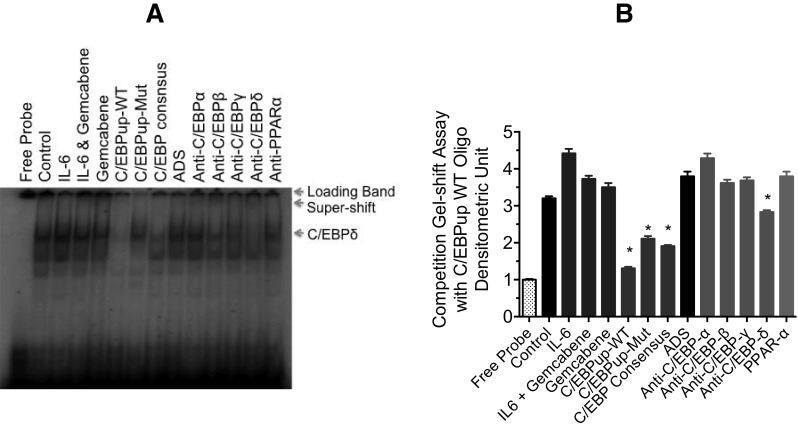
Characterization of the Binding Activity of C/EBPup-WT Oligonucleotides. Panel **A**, Nuclear extracts were prepared and gel shift assay performed as detailed in the “Materials and methods”. In competition assay, 100× cold oligonucleotides were used. Aldosterone synthase (ADS) oligonucleotide used as a non-specific control. For super-shift assay, various antibodies (400 ng) were added into reactions during pre-incubation time. Panel **B** Densitometric quantitation of polyacrylamide gel band in panel (**A**). The data presented show mean values of three experiments. All assays with no gemcabene contained 1% DMSO. *Indicates significant difference (*p* < 0.01)

The studies described above demonstrated that C/EBPdown promotor region is important for the gemcabene-mediated inhibition of CRP induction (Fig. 4A–D). Therefore, we sought to investigate the transcription factors responsible for gemcabene-mediated inhibition of IL-6-induced CRP promoter activity by using both the up-mutation and down-mutation of C/EBP promoter. First, we investigated the up-mutation construct since this promoter sequence did not show involvement in the gemcabene-mediated CRP promoter activity inhibition (Fig. 4B). For this, we prepared a double-stranded radiolabeled C/EBPup mutant and wild-type oligonucleotides to probe nuclear extracts isolated from Alexander human hepatoma cells, untreated or treated with IL-6 for 4 h (Fig. 5A, first 4 lanes). Various unlabeled double-stranded (ds) oligonucleotides or antibodies were used to characterize the interactions (Fig. 5). As shown in Fig. 5A, C, the top band (one of the two bands in the middle) is much stronger than the bottom band. IL-6-treated samples showed a much stronger top band compared to untreated control sample, while gemcabene reduced this IL-6-induction significantly (Fig. 5A, B): this band is indicated by an arrow. Cold C/EBPup wild-type completely competed away all of the bands (Fig. 5A), while C/EBP up-mutation showed a partial competition. C/EBP consensus oligo abolished almost all of the binding especially for the major band, whereas a non-specific ds-oligonucleotide, aldosterone synthase (ADS), had no effect (Fig. 5A). These results are consistent with the interpretation that those bands represent specific DNA–protein interactions and that they likely represent binding by one or more C/EBP isoforms. The interactions were further characterized using antibodies against the C/EBP isoforms indicated in Fig. 5. Anti-C/EBP-α antibody had no effect on any of the interactions. Addition of either anti-C/EBP-β or anti-C/EBP-δ antibodies resulted in somewhat decreased prevalence of bands (Fig. 5A) but were statistically significant only with C/EBP-δ antibody (Fig. 5B). A non-related antibody, anti-PPAR-α, showed no effect on either band. The quantitation of the bands indicates that IL-6 treatment increases C/EBP-β and -δ binding activity in Alexander human hepatoma cells (Fig. 5A). These findings also suggest that the C/EBPup mutation was not responsible for the gemcabene-mediated inhibition of CRP promoter activity, which is consistent with the cell-based studies with various mutant constructs (Fig. 4A, B). The involvement of C/EBPup sequence in the gemcabene-mediated inhibition would have shown blunted response, but it in fact showed significant response (Fig. 5A, B).

### Identification of proteins from nuclear extracts prepared from Alexander human hepatoma cells that interact with C/EBPdown

**Fig. 6:**
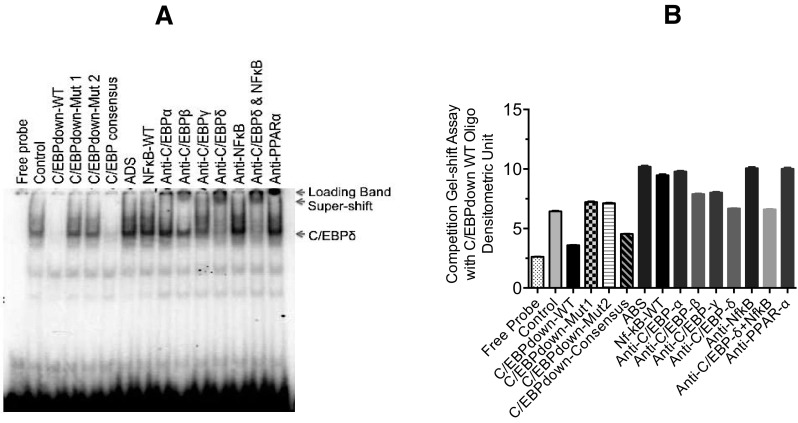
Characterization of the binding activity of C/EBPup-down-WT oligonucleotides. Panel **A** Characterization of the binding activity of C/EBPdown-WT oligonucleotides. Nuclear extracts were prepared and gel shift assay performed as detailed in “Materials and methods” section. In competition assay, 100× cold oligonucleotides were used. Aldosterone synthase (ADS) oligonucleotide used as a non-specific control. For super-shift assay, various antibodies (400 ng) were added into reactions during pre-incubation time. Panel **B** Densitometric quantitation of polyacrylamide gel band in panel **A**. The data presented show mean values with SEM of triplicate experiments. The SEM are so low that it is not visible in the bar graphs. All assays with no gemcabene contained 1% DMSO

Since the C/EBPup mutation was not responsible for the gemcabene-mediated inhibition of binding of transcription factor to C/EBP promoter (Fig. 5A, B), we sought to investigate if C/EBPdown sequence was responsible for the inhibition of gemcabene-mediated binding of the transcription factor to the C/EBP promoter. This appears somewhat plausible in view of the C/EBPdown sequence shown to be involved in the gemcabene-mediated inhibition of IL-6-induced promoter activity in the transfected Alexander human hepatoma cells (Fig. 4B). The double-stranded radiolabeled C/EBPdown oligonucleotide was used to probe nuclear extracts from Alexander cells, untreated or treated with IL-6 for 4 h. Various unlabeled double-stranded oligonucleotides or antibodies were also used to characterize the interactions (Fig. 6A, B). As shown in Fig. 6A, IL-6-treated nuclear extract samples showed a much stronger band compared to untreated control, suggesting induction of binding of transcription factor to the C/EBP promoter. This induction of transcription factor binding by IL-6 to C/EBP promoter sequence was found to be strongly competed away by cold C/EBPdown wild-type oligos as well as C/EBP consensus sequences (Fig. 6A, B), but not by the cold C/EBPdown mutant 1 and mutant 2 oligos, suggesting C/EBPdown sequence to be important in the binding of transcriptional factors to induce CRP transcription by IL-6 (Fig. 6A). The interactions were further characterized using antibodies against the C/EBP isoforms indicated in Fig. 6A, B. Anti-C/EBP-α and anti-NF-κB (p50) had no effect on any of the interactions. Addition of anti-C/EBP-γ resulted in a decreased prevalence of band although a clear super-shift was not seen (Fig. 6A). Anti-C/EBP-δ antibody did reduce binding to the promoter as evidenced by the disappearance of a band on the gel shift blot accompanied by the appearance of a super-shifted band, suggesting the importance of C/EBP-δ in CRP transcription. A non-related antibody, anti-PPAR-α, showed no effect on the intensity of either band. Furthermore, in order to distinguish that these super-shifts are not caused by non-specific interaction between the antibodies and gel shift oligonucleotides, the same set of antibodies for C/EBP and PPAR-α were also used in the gel shift assay with STAT3-CRP oligonucleotide. None of them showed an influence on the STAT3-CRP binding activity (data not shown). These results indicate that IL-6 treatment increases C/EBP-β binding to some extent while C/EBP-δ binding activity to a great extent in Alexander human hepatoma cells (Figs. 5A, 6B), demonstrating that the transcription factor C/EBP-δ is important in IL-6-induced inhibition of C/EBP promoter activity in Alexander human hepatoma cells.

## Discussion

In both the current preclinical studies and clinical studies, gemcabene has shown reduction of CRP [31], therefore the aim of the present study was to evaluate mechanism(s) resulting in gemcabene-mediated reduction of CRP. First, we evaluated gemcabene-induced regulation of CRP production in human hepatoma cells and anti-inflammatory activity in human coronary aortic endothelial cells, both important in the regulation of genes that influence progression of CVD. Our results show that gemcabene inhibited cytokine-stimulated CRP production in human hepatoma cells at doses consistent with clinical exposures. These data provide evidence that gemcabene reduction of CRP in patients [31] may occur via the mechanisms we observed in cells. We found that gemcabene interferes with IL-6 and IL-1β mediated induction of CRP production, suggesting that the compound has the potential to influence one or more steps in the signaling pathways induced by these cytokines. There appears to be several possibilities that may influence the signal pathways, including inhibition of phosphorylation and activation of Jak2, STAT3, or other signaling components of the IL-6 pathway [33, 43]. Acute coronary syndrome patients have elevated inflammation as a result of increased CRP in coronary circulation [27]. Since dissociation of pentameric to monomeric C-reactive protein releases CRP in circulation [28] and formation of CRP and lysophosphatidylcholine complex reduces the proatherogenic effects of macrophages [48], the antiatherogenic property of gemcabene appears to be responsible for the attenuation of interplay between CRP, atherogenic LDL, and atherosclerosis [26].

Our investigation into the mechanism of gemcabene inhibition of CRP secretion suggested several steps where the drug could act, including gene transcription, mRNA stability, protein stability, or secretion. Although not shown here, we tested the effects of several statins in the same in vitro system and found that none of them, even at very high concentrations, caused modulation in the secretion of CRP. This not only reinforces the specificity of the observations with gemcabene, but also suggests that, while statins may not influence IL-6 signaling directly, they may inhibit IL-6 production by mechanisms similar to those reported with human monocytes and rat vascular smooth muscle cells [49]. Statins function primarily by inhibiting cholesterol synthesis via inhibition of the HMG-CoA reductase enzyme and ultimately lowering LDL formation [50]. Thus, the primary mechanism of action of statins appears to be mainly through lowering LDL that ultimately reduces oxidized LDL in circulation [51] leading to attenuation of inflammation and reduction in CRP [52]. Therefore, the effect of statins on circulating CRP is indirect.

Plasma LDL chromatographically separates into five subfractions on the basis of its surface electric charge; L5 being the most electronegative subfraction, and the only one capable of inducing monocyte–endothelial cell adhesion [53]. Since statins lower L5 [54], and also reduce plasma levels of CRP [52], studies by Chu et al. [54] suggest that the plasma CRP and L5 reduction by statins occurs via lectin-like oxidized LDL receptor-1 (LOX-1) since both CRP and L5 are ligands for Lox-1 [54]. Indeed, statins inhibited Lox-1 expression in atheroma of WHHL rabbits, an animal model of HoFH, [55]. It was reported [54] that L5 dramatically increased the secretion of CRP in HAECs. In contrast, L1 had no effect on endothelial cell morphology or CRP expression. Thus, one of the mechanisms of CRP lowering by statins appears to be through reduction of electronegative LDL, L5, leading to lowering of CRP levels in plasma. Other studies demonstrate that IL-6 induced inhibition of CRP secretion by pravastatin, simvastatin, and atorvastatin in hepatocytes occurs via inhibition of STAT3 phosphorylation at Ser residue [56]. Gemcabene on the other hand lowers plasma CRP by a distinct mechanism through binding of transcription factor C/EBP-δ to the C/EBP response element in the proximal promoter of CRP (Figs. 4, 5, 6), suggesting that transcriptional regulation of CRP by gemcabene may be the primary mechanism of gemcabene-mediated lowering of CRP. Since gemcabene also lowers LDL in humans [31] and in animal models [29], and LDL lowering is linked to circulating levels of CRP [23, 31], it is possible that LDL lowering may have also contributed to CRP lowering in gemcabene-treated individuals. Thus, the primary mechanism of CRP reduction of these two lipid-lowering agents, atorvastatin and gemcabene, occurs via two different mechanisms. This is further corroborated by gemcabene’s efficacy in animal models of inflammation [57]. However, it should be noted that gemcabene and statins share a common property of lowering LDL, which may lead to reductions in plasma level of CRP [23, 31]. Thus, gemcabene lowers CRP by two mechanisms: one direct mechanism through down-regulation of CRP gene, and the other indirect mechanism though lowering of LDL. This is interesting in light of the possible gemcabene/statin combination therapy [31]. Due to the action of these compounds at different levels of the CRP response, it may be expected that combination therapy may result in an additive reduction of plasma CRP levels that occurs by different mechanisms. Indeed, clinical studies showed that statin and gemcabene combination therapy had additive effect on the reduction of circulating CRP [31].

We demonstrate here that gemcabene inhibits the secretion of IL-6 from HCAEC in a concentration-dependent manner. This is the first demonstration showing gemcabene’s anti-inflammatory activity in cells lining the coronary arteries. This finding is also consistent with the anti-inflammatory effect of this compound in human hepatoma Alexander cells. At this point, it is unknown whether or not these activities occur by the same mechanism. However, the anti-inflammatory effect on CRP expression in Alexander cells appears to be mediated at the transcriptional level through a C/EBP-dependent mechanism (Fig. 3A). It is noteworthy that the transcriptional control region of the IL-6, as well as the IL1-β promoter also contains C/EBP DNA recognition sequences [33, 38, 46]. We therefore used these cytokines to induce CRP promoter activity to study gemcabene-mediated inhibition in Alexander hepatoma and endothelial cell lines. Reduction of IL-6 in endothelial cells can possibly reduce the insult to the artery wall and thereby reduce the progression of atherosclerotic plaque formation [58, 59]. In a separate study, we have shown that gemcabene lowers CRP mRNA in human hepatocytes (data not shown), further supporting our conclusion that the gemcabene-mediated lowering of CRP proteins occurs at the transcriptional level.

The results presented in this report demonstrate that gemcabene can also inhibit IL-6-induced activity of the human CRP promoter (Fig. 3B). Further, it demonstrates that the downstream C/EBP binding site is required for the inhibition. It is clear from these results that the two C/EBP binding sites examined here are functionally distinct in that the upstream site is not as strongly influenced by gemcabene. One notable difference between the sites is the overlap between the downstream site and an NF-κB binding site. The previously described [34] NF-κB mutation used in the current study blunted gemcabene inhibition in both the wild-type and upstream mutant promoters, suggesting that NF-κB plays a role in the gemcabene-mediated effect. The results of inhibition of IL-6 and IL-1β-induced CRP production in the present study and the NF-κB-mediated induction of these cytokines [34] suggest that gemcabene can inhibit CRP gene transcription by interfering with the NF-κB-mediated IL-6/IL-1β pathway. This interpretation is consistent with the anti-inflammatory properties ascribed to gemcabene in studies described above. In addition, it is consistent with the hypothesis that gemcabene turns on a set of genes similar to that observed with a lipid-lowering agent [34]. It is also noteworthy that the STAT site, at positions − 216 to − 209 (Site 3), that contains a consensus STAT 3 response element [47], influences the level of IL-6-induced expression, but does not appear to play a role in the gemcabene-mediated inhibition.

Further investigation revealed that C/EBP-δ is one of the transcription factors that binds to the promoter of CRP. The interaction between C/EBP and CRP is modulated by gemcabene via interfering with the binding activity. It is clear that C/EBP-δ plays a major role in this inducible transcriptional regulation. Both C/EBP upstream and downstream binding sites are involved in this gemcabene-modulated DNA binding. Although the NF-κB binding site in CRP promoter is partially overlapped with C/EBP downstream binding site (6 nucleotides), we did not find any significant change on gemcabene effect with an NF-κB antibody. This is likely due to lack of sufficient binding site.

Gemcabene clearly shows anti-inflammatory properties in a number of cell-based study results. This suggests that gemcabene should be able to attenuate disease severity in animal model of inflammatory disease. Indeed, we have carried out *in vivo* studies in a number of animal models of inflammatory disease and found dose-dependent attenuation of disease severity [57]. Further evidence that anti-inflammatory agents lower CRP comes from human studies [60], which showed improvements in CVD risk [61].

**Fig. 7:**
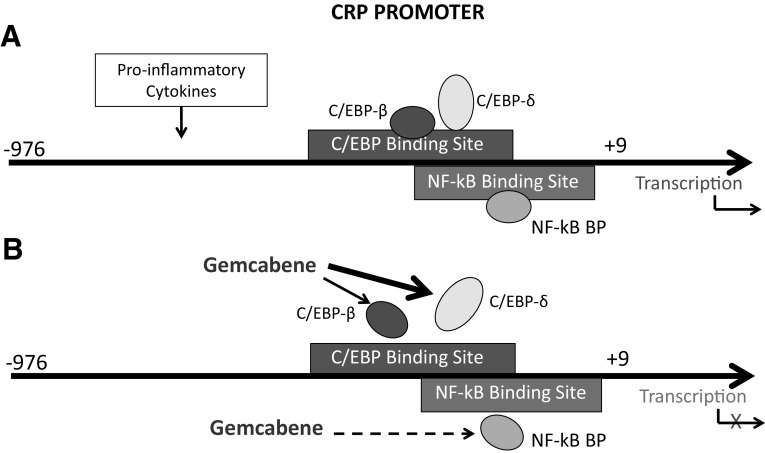
Mechanism of gemcabene-mediated inhibition of CRP transcription. Proinflammatory cytokines (IL-6/IL-1β) induce transcription of CRP through CRP promoter having sequences that bind to transcription factors C/EBP and NF-κB. Gemcabene interferes with the binding of C/EBP-δ to its promoter sequence to a great extent and binding of C/EBP-β and NF-κB to some extent. Together, the gemcabene’s interference with the binding of transcription factors to its cis-acting sequences leads to the inhibition of CRP transcription. The major player in gemcabene-mediated inhibition of CRP transcription is C/EBP-δ, and minor players in the inhibition of CRP transcription are C/EBP-β and NF-κB

Based on our present results, we suggest the working hypothesis shown in Fig. 7. Proinflammatory cytokines induce CRP transcription and increase the production of CRP. The presence of gemcabene inhibits cytokine-induced binding of C/EBP-δ as well as NF-κB to the C/EBP promoter and inhibits CRP transcription. This in turn reduces the production of CRP. Additionally, gemcabene may reduce plasma levels of CRP indirectly through reductions in LDL, which suggests the possibility that gemcabene blocks proinflammatory cytokine action in the liver by transcriptional mechanism (Fig. 7) and may explain the efficacy of this compound in reducing CRP levels in clinical trials.

A just competed clinical trial, CANTOS (Canakinumab Anti-inflammatory Thrombosis Outcomes Study) [62], with anti-inflammatory agent further confirmed the benefits of anti-inflammatory agents in attenuating cardiovascular events. Inhibition of IL-1β-induced inflammation and CRP production by gemcabene in the present study and improvements in CVD events by an inhibitor of IL-1β, canakinumab, in CANTOS study, suggest that anti-inflammatory agents may improve CVD events. Thus, gemcabene’s anti-inflammatory property, in addition to LDL lowering activity, offers added benefit to CVD patients.

## Abbreviations

CRP: C-reactive protein
C/EBP: CCAAT-enhancer-binding protein
HAEC: Human aortic endothelial cell
HCAEC: Human coronary aortic endothelial cells
HPMC: Hydroxypropyl methylcellulose
ICAM-1: Intercellular adhesion molecule-1
IL-1β: Interleukin-1β
IL-6: Interleukin-6
IL-8: Interleukin-8
Jak2: Janus kinase-2
JUPITER: Justification for the use of statins in primary prevention: an intervention trial evaluating rosuvastatin
Lox-1: Lectin-like oxidized low-density lipoprotein receptor-1
MCP-1: Monocyte chemoattractant protein-1
NF-κB: Nuclear factor-κB
PGE2: Prostaglandin E2
STAT: Signal transducer and activator
TNF-α: Tumor necrosis factor-α
VCAM1: Vascular cell adhesion molecule-1
WHHL: Watanabe homozygous hyperlipidemic
